# Genome-Wide Identification and Characterization of the *AlkB* Gene Family in Sweet Orange (*Citrus sinensis*)

**DOI:** 10.3390/cimb45010009

**Published:** 2022-12-26

**Authors:** Aijun Huang, Ying Wang, Peipei Gu, Zhixun Yang, Junna Han, Long Yi

**Affiliations:** 1 College of Life Scinece, Gannan Normal University, Ganzhou 341000, China; 2 National Navel Orange Engineering Research Center, Ganzhou 341000, China

**Keywords:** sweet orange, AlkB homolog proteins, ‘*Candidatus* Liberibacter asiaticus’

## Abstract

Sweet orange (Citrus sinensis) is a sub-tropical fruit crop with important economic value that is popular worldwide; however, various pathogens significantly affect citrus cultivation and distribution. AlkB homolog (ALKBH) proteins play crucial roles in RNA metabolism and translation in plants; however, no systematic investigations have been performed on ALKBH in sweet oranges. In this study, ten ALKBH gene family members were identified in Citrus sinensis genome. Standardized analyses, including physical properties, phylogenetic analysis, gene structure, motif composition, cis-acting element prediction, chromosome distribution, and synteny analysis, were conducted. The phylogenetic analysis suggested that the ten proteins were clustered into three groups, each of which had similar motifs and gene structures. Gene expression profiling revealed that almost all CsALKBH proteins were highly expressed in callus, and ALKBH9/10-like group members responded positively to biotic stress. Overall, this study is the first to report a genome-wide assessment of the ALKBH family in sweet oranges and provides valuable insights for candidate gene selection and elucidating the molecular mechanism of sweet orange response to pathogenic infections.

## 1. Introduction

AlkB homolog (AlkB homolog, ALKBH) proteins are a family of specific demethylases of the dioxygenase superfamily; they use cellular, molecular oxygen to oxidize the alkyl groups, predominantly methyl substituents, on alkylated nucleic acid bases, such as N6-methyladenine (m^6^A), 3-methylcytosine (M^5^C), and 1-methylguanine (m^1^G), to regenerate natural nucleotides, subsequently releasing the oxidized alkyl group as aldehydes and carbon dioxide [1]. They are present in almost all cellular organisms, except archaea and yeast, and play an important role in RNA metabolism and translation control, thus affecting various biological functions, such as growth, development, and stress response, among others [2,3].

To date, many ALKBH proteins have been identified in different plants and associated with various cellular roles [4]. In *Arabidopsis thaliana*, AtALKBH10B demethylated m^6^A-based messenger RNA (mRNA) modifications and mediated m^6^A demethylation of transcripts of key flowering time genes to specifically regulate the floral transition process [5]. AtALKBH9B demethylated m^6^A in the viral RNA of alfalfa mosaic virus (AMV) through specific interactions with AMV coat protein and positively regulated AMV accumulation and systemic invasion [6]. AtALKBH1D functioning as a demethylase in the chloroplasts is essential for transfer RNA (tRNA) biogenesis [7]. AtALKBH6 plays a role in seed germination and post-germination seedling growth under abiotic stress [8]. In Populus, PagALKBH9B and PagALKBH10B mediate m^6^A RNA demethylation and play regulatory roles in poplar growth and development. Their overexpression elevate transgenic line salt tolerance but reduce tolerance to drought stress due to decreased proline content [9]. A recent investigation demonstrated that SIALKBH2 could bind to the transcript of SIDML2, a DNA demethylase gene required for tomato fruit ripening, and modulate its stability via m^6^A demethylation, ultimately affecting fruit ripening [10].

The ALKBH gene family has been identified and characterized in *Arabidopsis thaliana*, *Solanum lycopersicum*, *Beta vulgaris*, *Populus nigra* [9,11,12,13]. Citrus is the largest economically important fruit crop worldwide. Sweet orange (*Citrus sinensis*) is a popular citrus variety, owing to its high nutritional value. Whole-genome sequencing of sweet oranges promotes the comprehensive analyses of numerous known gene families. However, information on the identification and functional characterization of ALKBH proteins in citrus is limited. Therefore, this study presents the first detailed and comprehensive description of the ALKBH gene family in the whole genome of sweet oranges. Ten putative CsALKBH genes were identified and their gene characteristics were analyzed. The phylogenetic relationships between *C. sinensis* and *A. thaliana* were identified, and RNA-Seq and qRT-PCR analyses were performed to confirm the tissue expression patterns. This study provides details of the ALKBH gene family in sweet orange to facilitate further functional characterization.

## 2. Results

### 2.1. Identification of ALKBH Gene Family Members in C. sinensis

Ten ALKBH proteins were identified based on the HMM search. These proteins were designated CsALKBH1 to CsALKBH10 based on their homology with *A.thaliana* ALKBH proteins; their detailed characteristics, including the number of amino acids, molecular weight, isoelectric point, instability index, aliphatic index, GRAVY, and subcellular localization, are listed in Table 1. The deduced protein sequences of the ten CsALKBH transcript lengths ranged from 195 to 913 aa, and the molecular weights ranged from 22.27 to 102.33 kDa. Six proteins with ˂6.5 pI values were acidic, three with ˃7.5 pI values were alkaline, and one with pI value between 6.5 to 7.5 was neutral. They were mostly predicted to be unstable because nine of them, except for CsALKBH2, had instability index values higher than 40. The aliphatic indexes of CsALKBH proteins were all between 70.01 to 95.90, and GRAVY values ranged from −0.754 to −0.172, indicating that all CsALKBHs were hydrophilic. The subcellular localization prediction findings indicated that nine CsALKBH proteins were located in the nucleus and one in the chloroplast.

**Figure 1:**
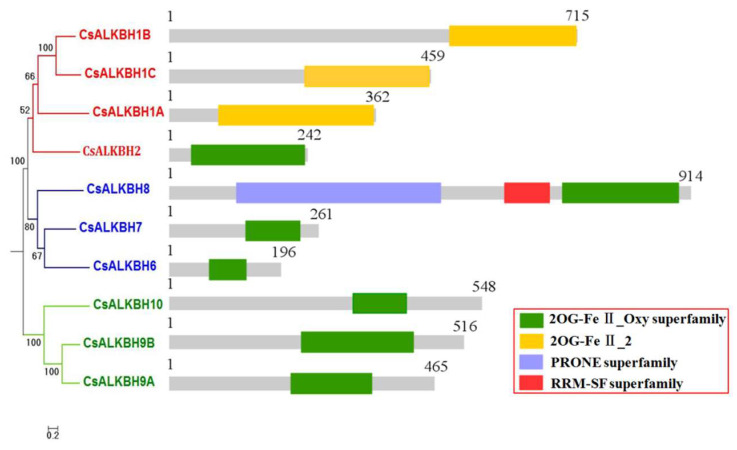
Conserved domain analysis of CsALKBH proteins. Domains are represented by different colored boxes.

The domains of the ten candidate CsALKBH proteins were analyzed by CCD. All proteins had 2OG–Fe–Ⅱ Oxy superfamily or 2OG–Fe–Ⅱ_2 domain. CsALKBH1A, CsALKBH1B, and CsALKBH1C had 2OG–Fe–Ⅱ_2 domain, while the rest of the CsALKBHs had 2OG–Fe–Ⅱ Oxy superfamily domain, indicating that these domains are highly conserved. Regarding domain distribution, CsALKBHs domains were located at the C-terminus (Figure 1). The RNA recognition motif (RRM) domain of CsALKBH8 was related to mRNA and rRNA processing, RNA output, and RNA stability by query. The plant-specific Rop nucleotide exchanger (PRONE) domain of CsALKBH8 was related to the guanine exchange factor (GEF) of the plant Rho GTPase [14].

### 2.2. Phylogenetic Analysis and Classification of CsALKBHs

**Figure 2:**
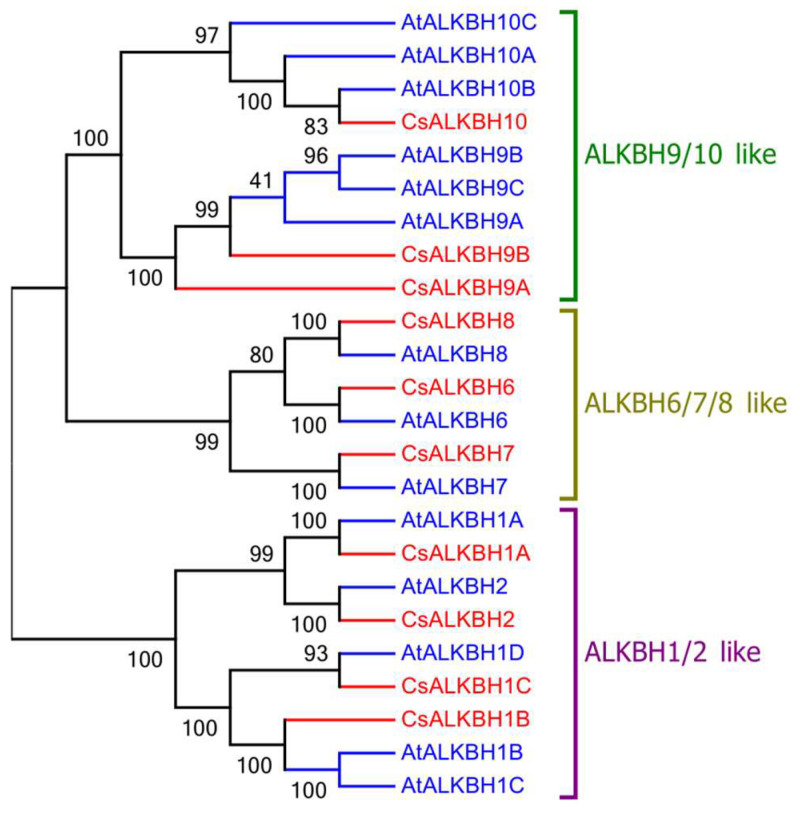
Phylogenetic analysis of CsALKBH proteins among *Citrus sinensis* (red) and *Arabidopsis thaliana* (blue), the tree was divided into five clades, which are marked by different colors and named as ALKBH9/10-like, ALKBH6/7/8-like, and ALKBH1/2-like.

To fully elucidate the evolutionary relationship between CsALKBH proteins, a phylogenetic tree was constructed with the 10 identified CsALKBHs and 14 reported AtALKBHs of *A. thaliana* using neighbor-joining method. The results showed that most bootstrap values were greater than 80, indicating high reliability. The 24 proteins were clustered into three groups: ALKBH 9/10-like, including CsALKBH9A, CsALKBH9B, and CsALKBH10; ALKBH 6/7/8-like, including CsALKBH6, CsALKBH7, and CsALKBH8; and ALKBH 1/2-like, including CsALKBH1A, CsALKBH1B, CsALKBH1C, and CsALKBH2 (Figure 2). The phylogenetic tree revealed that the ALKBH gene family was relatively evolutionarily conserved.

### 2.3. The Chromosomal Location of CsALKBHs

**Figure 3:**
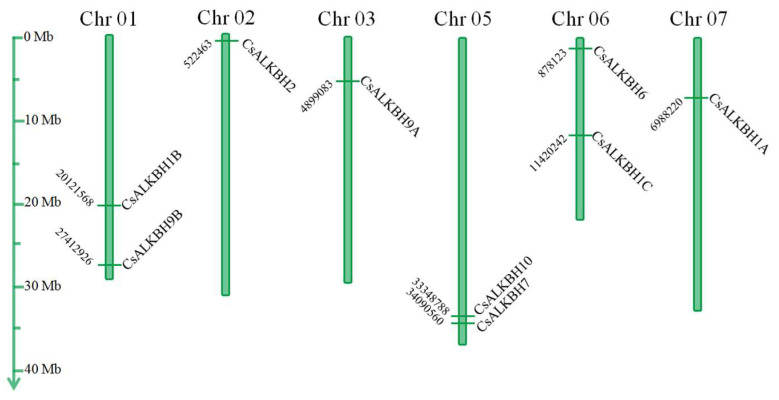
Distribution of CsALKBH genes on chromosomes. The leftmost scale shows chromosome length. Chromosomes are represented by green bars.

Chromosomal location analysis showed that ten CsALKBH genes were distributed on six of the nine *C. sinensis* chromosomes (Chr) (Figure 3), excluding Chr 04, Chr 08, and Chr 09. The number of CsALKBH genes on each chromosome ranged from one (Chr02, Chr03, Chr07) to two (Chr01, Chr05, Chr06). CsALKBH8 was located on unattributed scaffold. CsALKBH1A, CsALKBH1B, and CsALKBH1C were located on Chr 07, Chr 01, Chr06, respectively. CsALKBH2 and CsALKBH6 were located on Chr 02 and Chr 06, respectively. CsALKBH7 and CsALKBH10 were located on Chr 05. CsALKBH9A and CsALKBH9B were located on Chr 03 and Chr 01.

### 2.4. Gene Structural and Conserved Motif Analysis of CsALKBHs

**Figure 4:**
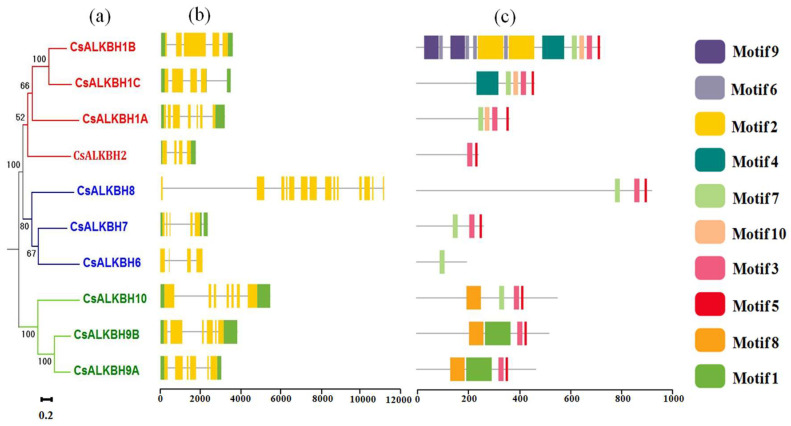
Phylogenetic tree, gene structure, and motif analysis of CsALKBHs. (**a**) The phylogenetic tree of CsALKBH is divided into three groups. (**b**) Exon–intron structure of CsALKBH genes. (**c**) Distribution of all motifs identified by MEME.

Given that pattern diversity of exon/intron structures and motifs plays an important role in the gene family evolution, the exon/intron structure patterns of CsALKBH genes were studied, and motifs were conserved based on their phylogenetic relationships (Figure 4a). Studies on exon/intron structure have shown that most members of the same group have similar exon/intron numbers but differ in length (Figure 4b). CsALKBH gene structure in each group was similar; however, differences were observed in the exon/intron arrangements of some genes. In particular, in group ALKBH 6/7/8-like, the 5′-end of CsALKBH8 had the longest non-coding region and its exons (14 exons) were almost triple that of CsALKBH7 (five exons) and CsALKBH6 (four exons). Neither CsALKBH8 nor CsALKBH6 contained non-coding regions. These results indicate that the CsALKBH genes were relatively evolutionarily conserved, ensuring gene structure integrity to limit changes in their function.

To identify common motifs among the different groups of CsALKBH proteins, the MEME motif search tool was used to identify ten conserved motifs. The proteins in the same group exhibited similar motif distribution patterns (Figure 4c). Motifs 3 and 5 were identified in all CsALKBH genes (except CsALKBH6). In addition to the common motifs, specific motifs were present in each group; motif 1 was only present in group ALKBH 9/10-like, and motifs 6 and 9 were only present in CsALKBH9. According to these results, the CsALKBH genes in the same subgroup had similar conserved motif compositions and distributions, suggesting that CsALKBH members in the same cluster may share similar functions.

### 2.5. Cis-Element Analysis of the CsALKBH Promoter in Citrus

**Figure 5:**
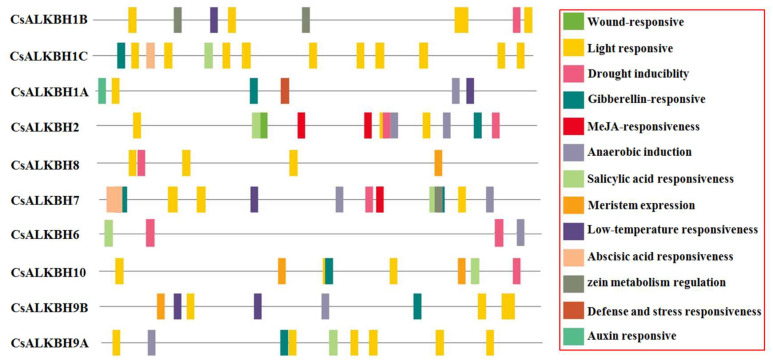
Putative cis-elements existed in the 1.5 kb upstream region of CsALKBH genes. The elements are displayed in different colored boxes. The homeopathic elements are represented by different colored boxes and their names and functions.

Abundant responsive regulatory elements were identified in CsALKBH promoter regions using PlantCARE analysis (Figure 5). The screened cis-elements were divided into two categories. The first type of element was hormone response, such as the gibberellin-responsive element, the MeJA responsiveness element, the salicylic acid responsiveness element, abscisic acid responsiveness element, and auxin responsive element. The second was the stress response, such as the wound response element, light responsive element, and the defense- and stress-responsive element. The light responsive element was identified abundantly in CsALKBH promoter regions, among which CsALKBH1C contained ten light responsive elements.

### 2.6. Synteny Analysis of CsALKBHs

**Figure 6:**
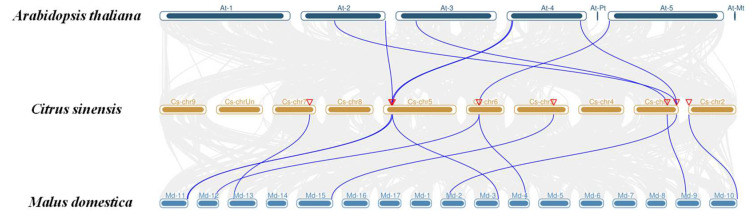
Collinearity analysis of ALKBH gene family between *Citrus sinensis* and representative species. The blue line indicates the collinearity of the ALKBH gene family in *Citrus sinensis* and the corresponding representative species. Other collinearity between genomes is indicated by gray lines.

A synteny analysis of the CsALKBH genes between *C. sinensis*, *A. thaliana*, and *M. domestica* was conducted (Figure 6). Five and eight orthologous pairs existed between *C. sinensis* and *A. thaliana*, and *C. sinensis* and *M. domestica,* respectively. CsALKBH9B and CsALKBH10 had two pairs of homologous genes in *A. thaliana*, whereas CsALKBH7, CsALKBH1C, and CsALKBH1B had one pair. CsALKBH1C and CsALKBH10 had two pairs of homologous genes in *M. domestica*, and CsALKBH1A, CsALKBH7, CsALKBH9A, CsALKBH9B, CsALKBH1B, and CsALKBH2 had one pair. Based on these results, it was speculated that *C. sinensis* and *M. domestica* might have a high homology.

### 2.7. Tissue-Specific Expression Profiles of CsALKBH Genes

**Figure 7:**
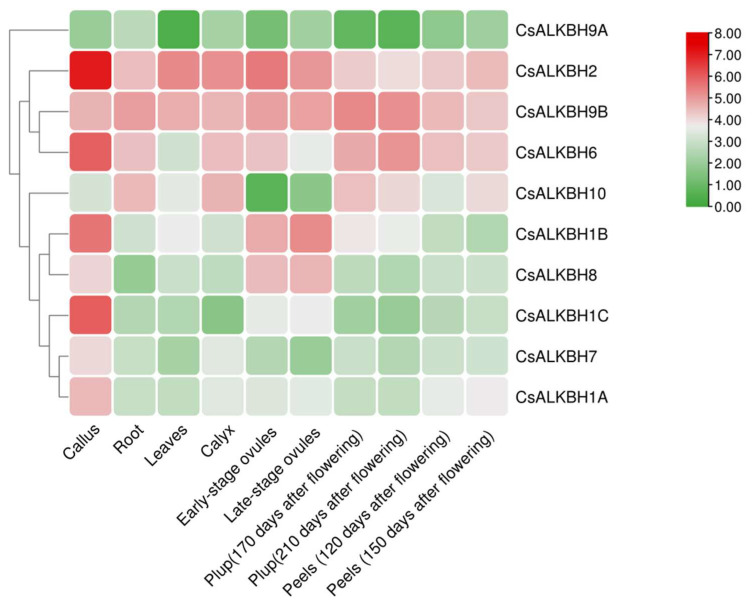
Expression patterns of CsALKBH genes in various *Citrus sinensis* tissues. A heatmap of CsALKBH RNA-seq data in ten tissues at different developmental stages was created by TBtools. The expression values mapped to a color gradient from low (green) to high expression (red) are shown at the right of the figure.

To further elucidate the role of CsALKBH genes in citrus growth and development, the temporal and spatial expression patterns of CsALKBH genes were analyzed using RNA-Seq data from different *C. sinensis* tissues (Figure 7). CsALKBH9A had low expression in all ten tissues, whereas CsALKBH2 was highly expressed. Almost all CsALKBH genes were highly expressed in callus, except for CsALKBH9A and CsALKBH10. Most of CsALKBH genes had medium expression levels in the leaves. In different tissues, CsALKBH2 and CsALKBH9B, as well as CsALKBH1A, ALKBH1C, and ALKBH7, exhibited similar expression patterns.

### 2.8. Change in Relative Expression of the CsALKBH Gene under Biotic Stress

**Figure 8:**
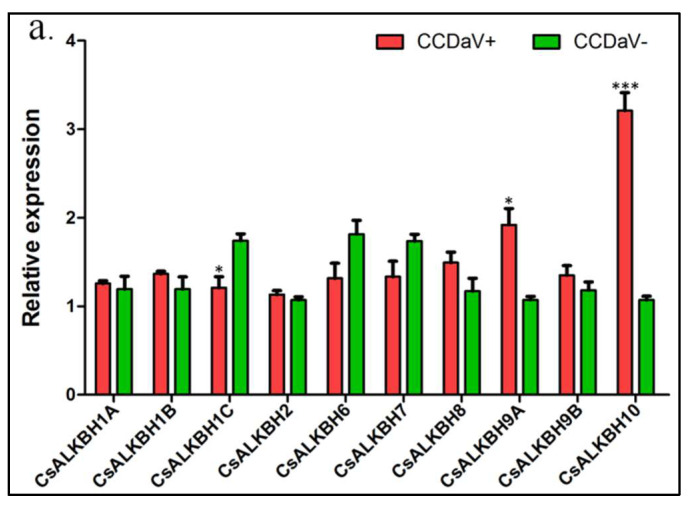

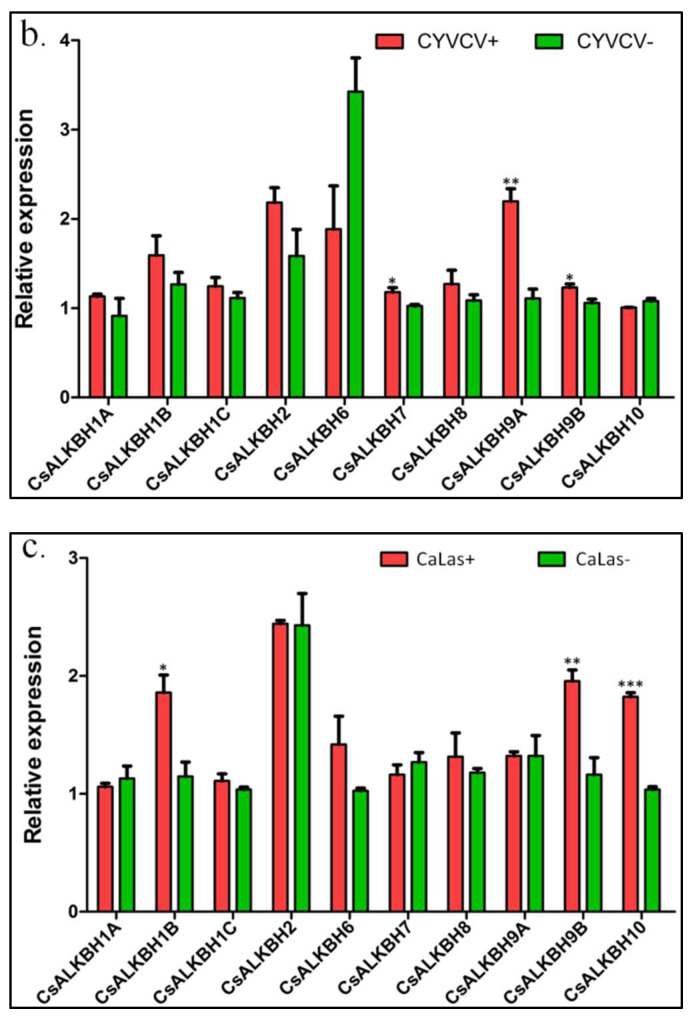
Expression profile of ALKBH genes on citrus in different biotic stress by qPCR analysis. (**a**), qPCR analysis expression of ALKBH genes in response to CCDaV; (**b**), qPCR analysis expression of ALKBH genes in response to CYVCV; (**c**), qPCR analysis expression of ALKBH genes in response to CaLas infection. Each value represents the mean ± SE of three replicates. * represents *p* ≤ 0.05, ** represents *p* ≤ 0.01, *** represents *p* ≤ 0.001.

Recent evidence has demonstrated that certain ALKBH genes are involved in plant response to infectious pathogens. In this study, three biotic stress treatments, CCDaV, CYVCV, and CaLas, were used to detect ALKBH genes response. CsALKBH9A, CsALKBH10, and CsALKBH1C expression levels were significantly affected in response to CCDaV infection. Of these, CsALKBH9A and CsALKBH10 were up-regulated; in particular, CsALKBH10 was up-regulated approximately threefold, whereas CsALKBH1C was down-regulated (Figure 8a). The expression levels of CsALKBH7, CsALKBH9A, and CsALKBH9B were significantly affected by CYVCV infection (Figure 8b). In response to CaLas infection, CsALKBH1B, CsALKBH9B, and CsALKBH10 were up-regulated approximately twofold (Figure 8c). Hence, the CsALKBH gene family members responded positively to biotic stress.

## 3. Discussion

RNA demethylation mediated by ALKBH genes functioning as eraser proteins is important in the epigenetic regulatory network for plant growth, development, and stress responses [4,8]. Yet the ALKBH gene family in *C. sinensis*, an economically important, globally recognized fruit crop, is yet to be studied. In the current study, through the genome-wide analysis, ten ALKBH family genes were identified. The number was similar to *Arabidopsis*, sugar beet, and tomato, but much less than quinoa, wheat, and populus, which may be attributed to being different copy numbers during plant evolution.

ALKBH genes in the *C. sinensis* genome varied significantly in their sequence structure. Regarding protein length, the variation range of the amino acid sequence of sweet oranges was 195 aa to 913 aa, whereas those of Arabidopsis, sugar beet, and tomato were 186 aa to 601 aa, 260 aa to 584 aa, 253 aa to 643 aa, respectively. The protein length of CsALKBH8 was 913 aa, almost double that of other homologous genes (AtALKBH8 431 aa, SIALKBH8 342 aa, and BvALKBH8B 487 aa). The variation range of the conserved motifs of *C. sinensis* was between 1 and 13, and some conserved motifs were unique to specific sequences or subgroups. These high variations in sequence structure revealed that CsALKBH gene family members acquired changes during evolutionary events that affected their functions.

Phylogenetic analysis is useful for identifying functional orthologous proteins. In this study, the ALKBH genes were divided into three groups, similar to previous studies. Among the groups, CsALKBH9A/9B and CsALKBH10 were homologs of AtALKBH9A/9B/9C and AtALKBH10A/10B/10C proteins, respectively, which showed m^6^A demethylation functions. This result suggest that CsALKBH9A/9B and CsALKBH10 may possess m^6^A demethylase activity. M^6^A RNA methylation, the most abundant chemical modification in eukaryotic cells [15], also occurs in viral RNAs [16]. Previous reports have supported its involvement in plant viral infections [6,17]. AtALKBH9B can affect m^6^A abundance in the viral genome of AMV and negatively affect AMV accumulation and movement in plants [6]. In this study, CsALKBH9A and CsALKBH10 were significantly affected by CCDaV infection and CsALKBH9A/9B was affected by CYVCV infection, these results suggesting that CsALKBH9A/9B and CsALKBH10 may be associated with these two viral infections; further research is required to confirm this association. In addition, CsALKBH9B and CsALKBH10 were also significantly up-regulated during CaLas infection.

Several cis-acting elements related to light, hormones, and stress responses were identified, suggesting that the ALKBH gene family has diverse functions in *C. sinensis* and may play a regulatory role in these physiological processes. Furthermore, ALKBH had a similar gene structure and conserved motifs in the same group, but differed significantly among the groups. Collinearity analysis was performed between *C. sinensis*, *A. thaliana*, and *M. domestica*. Five pairs of homologs were observed between *C. sinensis* and *A. thaliana*, whereas eight pairs were observed between *C. sinensis* and *M. domestica*, indicating that the evolution of the ALKBH gene was highly conserved and homologous.

## 4. Materials and Methods

### 4.1. Identification of ALKBH Gene Family in C. sinensis

The *C. sinensis* genome, protein sequences, and annotation files were downloaded from the Citrus Pan-genome to Breeding Database (CPBD, http://citrus.hzau.edu.cn/index.php, accessed on 2 May 2022) [18]. All proteins sequences from sweet orange genome were scanned by HMMER 3.0 using a hidden Markov model (HMM) file of the 2OG-Fe-Ⅱ-oxy domain (PF13532) downloaded from the Pfam website (http://pfam.xfam.org, accessed on 3 May 2022), with an E value of 1 × 10^–5^. For the second HMM search, the first sequences obtained were used as candidate members to rebuild a new HMM model. Candidate protein sequences were obtained based on the results of the two HMMER searches. The presence of the conserved domain of 2OG-Fe-Ⅱ-oxy domain in the predicted protein was verified using the NCBI Conserved Domain search (https://www.ncbi.nlm.nih.gov/Structure/cdd/wrpsb.cgi, accessed on 5 May 2022) and SMART program (http://smart.embl.de/smart/batch.pl, accessed on 5 May 2022). Protein length, molecular weight (MW), theoretical isoelectric point (pI), instability index, aliphatic index, and grand average of hydropathicity (GRAVY) were calculated by ExPASy (https://web.expasy.org/protparam/, accessed on 6 May 2022) [19]. Subcellular localization was predicted using an online analysis tool BUSCA (https://busca.biocomp.unibo.it/, accessed on 6 May 2022).

### 4.2. Alignment and Phylogenetic Analysis

Multiple alignments of selected full-length amino acid sequences were aligned with default parameters using ClastalW sequence alignment methods. Alignment results were used to construct a neighbor-joining (NJ) tree using MEGA 7.0 software with Poisson correction, partial deletion, and the bootstrap value set as 1000 (http://www.megasoftware.net/, accessed on 8 May 2022) [20]. The bootstrap values (>50%) of the major branches were revealed. An Interactive Tree Of Life (iTOL) v6 (https://itol.embl.de/, accessed on 10 May 2022) was used to beautify the phylogenetic tree.

### 4.3. Gene Structure, Conserved Motif, and Cis-Element Analysis

Information on gene length and structure was extracted from the sweet orange genome GFF file (CPBD, *Citrus sinensis* v1.0). The conserved motifs were scanned using MEME Suite software (http://meme-suite.org/tools/meme, accessed on 12 May 2022) [21], with a maximum motif number of ten and the remaining parameters set to default values; the output file (xml suffix) was downloaded. Genomic DNA sequences 1500 bp upstream of each CsALKBH gene start site were extracted from the sweet orange genome FASTA file and submitted to the PlantCARE database (http://bioinformatics.psb.ugent.be/webtools/plantcare/html/, accessed on 13 May 2022) [22] to identify cis-elements; the output file (tab suffix) was also downloaded. Finally, all downloaded files were submitted to TBtools software for visualization [23].

### 4.4. Chromosomal Location and Syntenic Analysis

The length of each chromosome and positional information of the CsALKBH gene on the chromosomes were extracted from the sweet orange genome GFF file using TBtools. MapChart 2 was used to map the position and relative distance of each CsALKBH gene on the chromosome. One-Step MCScanx was used to predict the synteny between the ALKBH genes in *C. sinensis*, *A. thaliana*, and *Malus domestica*, using genome annotation files and genome sequences files. The multiple synteny plot for MCScanX in TBtools software (version 1.098696) was used to visualize synteny. The genome data of *A. thaliana* and *M. domestica* were downloaded from the EnsemblPlants website (https://plants.ensembl.org/index.html, accessed on 15 May 2022).

### 4.5. Expression Patterns Analysis of ALKBH Genes by RNA-Seq Data

To study the ALKBH gene-specific expressions in different *C. sinensis* tissues, RNA-Seq data of different tissues and organs (callus, root, leaves, calyx, ovules, pulp, and peels) were downloaded from CPBD. The TBtools software was used to draw the expression heat map of ALKBH genes.

### 4.6. Plant Materials and Quantitative Real-Time PCR

Forty one-year old *C. sinensis* plants with similar developmental status were used for viral treatment. Budwoods from *Citrus chlorotic dwarf-associated virus* (CCDaV)-, *Citrus yellow vein clearing virus* (CYVCV)-, and ‘*Candidatus* liberibacter asiaticus’ (CaLas)-infected plants were grafted onto ten plants. Budwoods from healthy plant were grafted onto ten other plants, which were used as controls. All the plant materials were nurtured in the greenhouse of National Navel Orange Research Center, Ganzhou, China. For all treatments, plant materials from three biological replicates were immediately harvested, frozen in liquid nitrogen, and stored at −80 °C until RNA isolation.

Total RNA was extracted from *C. sinensis* leaves using an RNA-easy isolation reagent (Vazyme Bio Inc., Nanjing, China), according to the manufacturer’s instructions. RNA was treated with RNase-free DNase I to remove potential genomic DNA contamination, and its quality and quantity were assessed with a NanoDrop 2000c spectrophotometer (ThermoFisher Scientific, Waltham, MA, USA), followed by immediate storage stored at −80 °C for further analysis.

Complementary DNA (cDNA) was synthesized using the HiScript 1st strand cDNA Synthesis Kit (Vazyme Bio Inc., Nanjing, China), according to the manufacturer’s instructions. All cDNA samples were diluted to the same concentration as the RT-qPCR analysis template. Specific ALKBH gene primers were designed using Primer Premier 5; detailed information is provided in Table 2. RT-qPCR was conducted using the LightCycler 96 PCR detection system (Roche, Basel, Switzerland). The relative expression levels were calculated using 2^−∆∆Ct^ method [24]. The expression levels of the different sampling cycles were normalized with the F-box gene. GraphPad software was used for relative expressions analysis and completion of the relative expression histogram.

The volume of the reaction system was 20 μL, containing 2 μL cDNA template, 10 μL 2× SuperReal premix plus, 0.5 μL of 10 μM forward and reverse primers, and 7.0 μL of distilled deionized water. The amplification program conditions were as follows: 95 °C for 10 min, 40 cycles of 95 °C for 10 s, and 60 °C for 30 s. Each sample was replicated three times.

## 5. Conclusions

In this study, ten ALKBH gene members were identified in sweet orange genomes. These CsALKBH proteins varied, but all contained 2OG–Fe–Ⅱ Oxy superfamily or the 2OG–Fe–Ⅱ_2 domain. Phylogenetic analysis revealed that the identified CsALKBH genes could be classified into three groups, ALKBH1/2-like, ALKBH6/7/8-like, and ALKBH9/10-like. Members of the same group had similar gene structures and motif compositions. In addition, some CsALKBH genes had distinct expression specificities in different tissues and developmental stages. ALKBH9/10-like gene members were significantly regulated by virus or CaLas infection. Therefore, CsALKBH may be involved in multiple cross-regulated networks related to *C. sinensis* development and biotic stress responses. This study provides a foundation for further investigation of the functional properties of the ALKBH gene family in *C. sinensis*.

## Figures and Tables

**Table 1:** Detailed information of ALKBH genes family members in *Citrus sinensis*.

Genome ID	Gene Name	Protein Length (aa)	Molecular Weight (KD)	Isoelectric Point (PI)	Instability Index	GRAVY	Subcellular Localization
Cs7g10770	CsALKBH1A	361	40.83	6.12	55.08	−0.433	Nucleus
Cs1g16870	CsALKBH1B	714	78.26	6.54	44.41	−0.604	Nucleus
Cs6g09650	CsALKBH1C	458	51.33	9.11	53.01	−0.625	Chloroplast
Cs2g01630	CsALKBH2	241	28.22	9.53	31.97	−0.754	Nucleus
Cs6g01500	CsALKBH6	195	22.27	6.05	54.00	−0.172	Nucleus
Cs5g32950	CsALKBH7	260	29.61	4.46	50.05	−0.510	Nucleus
orange1.1t03596	CsALKBH8	913	102.33	6.25	55.40	−0.399	Nucleus
Cs3g04140	CsALKBH9A	464	51.92	8.52	52.44	−0.542	Nucleus
Cs1g25260	CsALKBH9B	515	57.87	6.46	47.36	−0.618	Nucleus
Cs5g31840	CsALKBH10	547	59.93	5.44	54.43	−0.340	Nucleus

**Table 2:** Primer sequences of CsALKBH genes.

Gene	Forward Primer (5′–3′)	Reverse Primer (5′–3′)
CsALKBH1A	TTCATACAATCAGAACGGTCA	CTTCCATACTTAACGCACCT
CsALKBH1B	AATATGAAACACCCCGAGT	TAAAACAATCATGGGCGAGA
CsALKBH1C	GCCGTCTGTTATTCCTTGTGA	AAGTATGTCTTCCACGTTGCT
CsALKBH2	TTAATTTACAGTGGCTACAGG	TTAAGCCAAATAGAAGAACTGA
CsALKBH6	CTTACAATGATTACGCGAAG	CATTATGCCTTGGTTAGGTT
CsALKBH7	ATAGATAACCCACATGCGGTA	CTGTTTGCGATTTATCTCGT
CsALKBH8	ATGGCTTCCGAATTCTACACCAG	TGCGAAATGTGAAAGATACCCT
CsALKBH9A	TTCCTTCTGATGATACCGAA	ATCCTGCTCTAGTGAACCTG
CsALKBH9B	TTTTCAAATCTGATGGCCTA	CACCCTCGACTTATCCGTA
CsALKBH10	TTCAGTGGCAACTAATACCAGA	TCACAAACAAGACTACGTCCA
F-box	TTGGAAACTCTTTCGCCACT	CAGCAACAAAATACCCGTCT

## Data Availability

All data are presented in the manuscript.
